# Pregnancy following laparoscopic hysteropexy—a case series

**DOI:** 10.1186/s10397-017-1017-1

**Published:** 2017-08-17

**Authors:** Helen Jefferis, Natalia Price, Simon Jackson

**Affiliations:** Department of Gynaecology, The Women’s Centre 8 John Radcliffe Hospital, Oxford University Hospitals NHS Trust 9, Headley Way, Oxford, OX3 9DU 10 UK

**Keywords:** Hysteropexy, Pregnancy, Fertility-preserving prolapse surgery

## Abstract

**Background:**

Uterine-preserving prolapse surgery offers the chance to retain fertility; however, limited data is available for the safety of pregnancy following surgery and the effect of pregnancy on surgical outcome. Our operative technique involves mesh encircling the cervix and uterine arteries, which raises concerns that compromise of uterine blood flow during pregnancy may lead to foetal growth restriction. We also think this necessitates delivery by caesarean section. We report on six pregnancy outcomes following laparoscopic hysteropexy. Primary outcomes were live birth and birth weight. Secondary outcomes were integrity of mesh and immediate effect on prolapse.

**Results:**

All patients had successful pregnancy outcomes with birth weights on or above the 10th centile. There was no effect on mesh integrity seen in any of the cases. There was no deterioration in apical prolapse when assessed post delivery, but two patients had new onset anterior vaginal wall prolapse.

**Conclusions:**

We think our technique of hysteropexy is safe for those wishing to conceive. Larger numbers are needed to allow robust evidence-based guidance for patients and clinicians.

## Background

Laparoscopic hysteropexy offers uterine preservation to patients with uterovaginal prolapse. It is the treatment of choice for patients wishing to retain fertility; however, limited data are available for pregnancy outcome following this surgery. Standard practice is to advise women to complete their family prior to any pelvic floor surgery, but in some cases, this may not be possible due to significant adverse effect on function and quality of life. In these cases, we need to be able to counsel women appropriately as to pregnancy outcome and whether further pregnancy is associated with recurrence of prolapse.

The technique previously reported from Oxford [1] entails complete cervical encirclage with polypropylene; a video describing this technique has been published [2]. A three or four port laparoscopic approach is used. The sacral promontory is dissected until a safe area of periosteum is identified. The peritoneum is then opened from this incision down to the right uterosacral ligament, keeping medial to the ureter. A flap of peritoneum is created at the level of the cervix to enable reperitonisation. The utero-vesical fold is opened and bilateral avascular windows were made in the broad ligament, lateral to the uterine arteries. Type 1 polypropylene mesh (ProleneTM mesh, Ethicon, Somerville, NJ, USA) is cut to a bifurcated shape; the arms are brought through the windows in the broad ligament, wrapped around the cervix and sutured to the cervix anteriorly. The mesh is completely reperitonised and transfixed to the sacral promontory using helical fasteners (ProtackTM, United States Surgical, Tyco Healthcare, Norwalk, CT, USA).The encirclage technique theoretically minimises the risk of mesh avulsing from the cervix, indeed we have not had a case of cervical avulsion in the 10 years we have been performing this surgery in Oxford (data not published). Complete encirclage as described above does, however, pose unique challenges in terms of future pregnancy. The mesh is placed lateral to the uterine arteries bilaterally. Compression of these could theoretically compromise blood flow to the utero-placental unit resulting in placental compromise and intrauterine growth restriction. It is also not known whether the mesh may inhibit or restrict formation of the lower uterine segment. In addition, the mesh encircles the cervix at the level of the internal os, preventing cervical dilatation and necessitating delivery by caesarean section. The impact of pregnancy on the mesh is also unknown—one concern would be the risk of a gravid uterus causing the mesh to avulse from the sacral promontory with subsequent recurrence of prolapse.

One case report has been published from our unit describing successful pregnancy outcome following this technique of laparoscopic hysteropexy [3]. Including this case, we now describe a series of six pregnancy outcomes, the largest data series in the literature to date. The primary outcome measures were live birth and birth weight. Secondary outcome measures were integrity of mesh and effect on prolapse.

## Methods

Six patients have presented to our department following laparoscopic hysteropexy with spontaneous conceptions. These cases were discussed at a multidisciplinary meeting between the obstetric and urogynaecology teams to plan antenatal care and delivery. Patients underwent uterine artery Doppler assessment at 22–23 weeks to evaluate whether blood flow had been compromised. Growth scans with umbilical artery assessment were performed at 28, 32 and 36 weeks gestation. All patients were delivered by caesarean section with a member of the urogynaecology team present to assess for mesh avulsion from the promontory. Patients were then seen at 8 weeks post delivery by a member of the urogynaecology team and were asked to subjectively describe any vaginal symptoms. They were examined and the POP-Q system was used to objectively assess prolapse.

One patient was not seen antenatally as she was not referred for consultant led care and was delivered as an emergency. She also did not attend her postnatal review. Ethics approval was not sought for this article as it is a retrospective case report.

## Results

All six cases resulted in live births with birth weight on or above the 10th centile (range 10–70th). The mesh remained attached to the sacral promontory in all cases. Follow-up took place at a median of 9 weeks post delivery (range 8–10 weeks). Two patients felt their prolapse was subjectively worse post delivery and objectively had developed new anterior compartment prolapse. No patient had any deterioration in apical support.

The six cases are described in detail below. Table 1 summarises pregnancy outcome data.

**Table 1:** Summary of pregnancy and outcome

	Patient 1	Patient 2	Patient 3	Patient 4	Patient 5	Patient 6
Age	41	42	42	28	39	27
BMI	30.1	18.7	25.6	23.0	17.8	24.9
Parity	3	2	2	2	2	3
Interval between surgery and delivery (months)	14	21	13	33	31	10
Antenatal care	Angulated right uterine artery, nil else	No concerns	No concerns	Back pain	No concerns	Midwifery led care
Delivery	Placenta praevia	Uncomplicated	Uncomplicated	Uncomplicated	Mesh over lower segment	Uncomplicated
Birth weight (grams)	3290	3520	3160	3335	3520	2795
Postnatal—prolapse worse?	Yes (cystocoele)	No	No	Yes (cystocoele)	No	DNA review

### Patient 1

In a 41-year-old para 3, laparoscopic hysteropexy was performed due to uterovaginal prolapse (Ba −1 cm, Point C −1 cm pre-operatively, Ba −3 cm, Point C −6 cm post operatively). She had thought her family was complete; however, she conceived spontaneously 7 months post-surgery and decided to continue with the pregnancy. Pregnancy care was undertaken as described above. Her uterine artery Doppler at 23 weeks showed significant right angulation of both arteries at the level of the internal os, presumably due the mesh; however, pulsatility index (PI) and resistance index (RI) of the uterine arteries was normal. Serial growth scans showed a normally grown foetus but a persistently low lying posterior placenta. For this reason, delivery was brought forward to 38 weeks. At caesarean section, the lower segment was well formed. The mesh was seen under the peritoneum and below the lower segment, well away from the lower segment incision.

Exploration of the abdomen at section confirmed the mesh remained securely attached to the sacral promontory. Her baby boy was of normal birth weight at 3290 g (50th centile).

At postnatal review, she felt that her prolapse symptoms were a little worse than before. On examination, the cervix remained well supported (C −6) as did the posterior wall (Bp −3), but there was anterior wall prolapse (Ba 0) and she has subsequently gone on to have an anterior repair with good anatomical result.

### Patient 2

A 42-year-old para 2 had a laparoscopic hysteropexy with posterior repair (Point C at 0 pre-operatively and −7 cm post operatively). She had a planned pregnancy with conception 1 year post surgery. Pregnancy care was as outlined above. Uterine artery Doppler and serial growth scans were normal. Caesarean section at 39 + 2 was uncomplicated, with a well-formed lower segment and no evidence of avulsion of the mesh. A baby girl was delivered with birth weight of 3520 g (70th centile). At postnatal review, she remained asymptomatic of prolapse, with the cervix remaining supported (C-7 cm).

### Patient 3

A 42-year-old para 2 had required IVF to conceive previously due to unexplained infertility. Following her second delivery, she underwent laparoscopic hysteropexy (pre-operatively point C + 1 cm, post-operatively −7 cm). Within 4 months of surgery, she spontaneously conceived (no contraception was being used due to the prior history of infertility and pregnancy was a surprise).

Uterine artery Doppler and growth scans were normal. Caesarean section was carried out at 39 + 1, with a well-formed lower segment and no evidence of mesh avulsion. A girl was delivered weighing 3160 g (40th centile). At postnatal review, no prolapse symptoms were reported and point C was at −6 cm.

### Patient 4

A 28-year-old para 2 had a laparoscopic hysteropexy for symptomatic prolapse (pre-operatively point C at 0, post-operatively at −7 cm, Ba −3 cm). Two years later, she conceived spontaneously and decided to continue with pregnancy. Antenatal care was as outlined previously, with normal uterine artery Doppler and serial growth scans. She did experience lower back pain from 16 weeks and attended hospital on two occasions with this but was managed with analgesia and physiotherapy. Caesarean section at 39 weeks was uncomplicated, the lower segment was well formed and the mesh remained attached to the sacral promontory. A girl was delivered weighing 3335 g (50th centile).

At postnatal review, she did report increasing vaginal symptoms of prolapse. On assessment, there was no change in uterine prolapse (Point C −7 cm) but there was a cystocoele not previously described (Ba −1 cm). At present, this is being managed conservatively.

### Patient 5

In a 39-year-old para 2, laparoscopic hysteropexy had been performed for uterine prolapse with point C + 2 cm (post-operatively −7 cm). The conception was 22 months following surgery and was planned. Antenatal care was as outlined above, with normal uterine artery Doppler and serial growth scans. During Caesarean section at 39 + 2 weeks, the mesh was felt over the lower segment and so a transverse hysterotomy was performed at the upper limit of the lower segment, resulting in a blood loss of 1000 mls. The mesh remained attached to the sacral promontory, and a baby girl weighing 3520 g (70th centile) was delivered.

At postnatal review, no prolapse symptoms were reported and point C remained −7 cm.

### Patient 6

A 27-year-old para 3 underwent laparoscopic hysteropexy with pre-operative findings of point C + 1 cm; post-operatively, this was corrected to −6 cm. She conceived 1 month following but was a late booker in pregnancy, first seeing her midwife at 18 weeks. Her booking history stated a background of “prolapse surgery” but no referral to consultant led care was made, and she therefore followed a low risk, midwifery led pathway of antenatal care with no additional scans. She presented to labour ward at 39 + 4 with ruptured membranes and was draining thick meconium. She was reviewed by the obstetrician on call who discovered the history of hysteropexy and therefore proceeded to emergency caesarean section.

This was uncomplicated, with a well-formed lower segment and no mesh avulsion. A baby boy was delivered weighing 2795 g (10th centile). All three of her previous children had birth weights on the 10th centile.

Unfortunately, this patient has not attended for a postnatal review.

## Discussion

Current advice to women is to complete their family prior to embarking on uterine-preserving prolapse surgery, due to the lack of knowledge about the impact of pregnancy on surgical outcome, and of any impact surgery may have on pregnancy. This series gives some limited data on the safety of encirclage mesh hysteropexy in women of childbearing age.

In the literature, two case reports of pregnancy following polypropylene mesh augmented sacrohysteropexy have been reported [4, 5]; however, this technique involved mesh transfixed to the posterior cervix only. The latter case experienced a recurrence of prolapse 2 years post delivery requiring repeat surgery. Pandeva et al. [6] report on eight pregnancy outcomes following a single sheet mesh sacrohysteropexy. Again, this technique does not encircle the cervix and so should not cause any alteration in uterine blood flow. One case report [7] describes a term vaginal delivery following an open sacrohysteropexy using a Y-shaped mesh transfixed anteriorly and posteriorly but not wrapping around the cervix.

The concerns regarding uterine artery compression with an encirclage technique appear to be unfounded; whilst one patient’s uterine artery Doppler showed some angulation of the artery, there was no change to the PI or RI in any cases. Growth was normal for all foetuses, with all women delivering babies with birth weights on or above the 10th centile. No woman developed pregnancy-related hypertension. This suggests that (in this small cohort at least) placental development and perfusion is not compromised by the hysteropexy mesh.

The lower uterine segment develops from the isthmus of the uterus, above the level of the internal os. At caesarean section, all but one woman had a normally developed lower uterine segment, meaning caesarean delivery was straightforward. In one case where mesh was noted over the lower segment, it is possible that the original surgery had left the “wrap around” portion of the mesh too loose, meaning it slipped above its usual position at the internal os. Our usual practice is to transfix the arms of the mesh tightly around the cervix to prevent slippage. In all cases, the mesh had remained fixed to the sacral promontory.

At postnatal follow-up, none of the women had any deterioration in apical prolapse; however, two women developed new onset symptoms and prolapse of the anterior compartment. At 8 weeks post-partum, the uterus has involuted to its usual size; however, patients may not have resumed full activity, making it harder to assess symptomatology. One patient has had further prolapse surgery in the form of an anterior colporrhaphy since her delivery. None of the other patients have re-presented with troublesome prolapse symptoms.

The main limitation of this study is the small sample size, meaning it is not possible to draw general conclusions about either foetal or maternal outcome. It is, however, the largest series to date and so offers some information for women and clinicians considering fertility-preserving prolapse surgery.

One concern for women undergoing pregnancy post hysteropexy is the question of how to manage pregnancy loss. Our belief is that in the first trimester, miscarriage could be managed as per usual protocols, as the cervical canal should admit a small suction curette. However, mid trimester and third trimester losses would result in the need for hysterotomy with the associated morbidity and impact on any future pregnancy.

The majority of women undergoing prolapse surgery will be past childbearing age. Our advice for the younger patients remains that they complete their family prior to reconstructive surgery. However, this will not always be possible; some younger women have significant prolapse impacting negatively on quality of life, and we need to be able to counsel them appropriately. All fertile women undergoing uterine preservation surgery in our unit are advised of the possible impact of pregnancy and if pregnancy is not desired should be offered reliable contraception at the time of surgery (in the form of a coil or salpingectomy). Those who are considering pregnancy should be advised that a caesarean section seems mandatory and that they should inform their GP or midwife at booking of the need for consultant led antenatal care.

## Conclusions

This study suggests that the Oxford Hysteropexy is suitable for women who wish to conceive. The hysteropexy does not appear to have an adverse effect on foetal growth, and delivery by caesarean is feasible. Furthermore, pregnancy does not appear to compromise long-term hysteropexy uterine support. In two out of the five patients assessed in the post-partum, there was de novo anterio vaginal wall prolapse. However, the study is small and further patient numbers are required before we can give patients robust evidence-based guidance as to the safety of pregnancy following hysteropexy. As these cases are uncommon, it would be sensible for units to pool any outcome data in the form of a national registry and database.
